# Ovarian tissue cryopreservation for a 3-year-old girl with Mosaic Turner syndrome in China: First case report and literature review

**DOI:** 10.3389/fendo.2022.959912

**Published:** 2022-11-21

**Authors:** Jiaojiao Cheng, Xiangyan Ruan, Juan Du, Fengyu Jin, Muqing Gu, Yurui Wu, Alfred O. Mueck

**Affiliations:** ^1^ Department of Gynecological Endocrinology, Beijing Obstetrics and Gynecology Hospital, Capital Medical University, Beijing Maternal and Child Health Care Hospital, Beijing, China; ^2^ Department for Women’s Health, University Women’s Hospital and Research Center for Women’s Health, University of Tuebingen, Tuebingen, Germany; ^3^ Department of Thoracic Surgery and Surgical Oncology, Children’s Hospital, Capital Institute of Pediatrics, Beijing, China

**Keywords:** ovarian tissue cryopreservation, fertility preservation, ovarian function, turner syndrome, premature ovarian insufficiency, follicle density

## Abstract

**Background:**

Although it cannot be predicted accurately which young females will develop premature ovarian insufficiency (POI) following chemotherapy or irradiation, patients at high risk of POI should be offered ovarian tissue cryopreservation (OTC). Our ovarian tissue cryobank is the first center in China. OTC was firstly performed on a 3-year-old girl with mosaic Turner syndrome (TS) in China. We report this case and present a literature review about TS girls’ fertility preservation (FP).

**Case presentation:**

Karyotype analysis of umbilical cord blood showed that the girl was diagnosed with TS, 45,X [19]/46,XX [81]. The girl was a 3-year-old girl when her parents would like OTC to preserve fertility. No abnormality was found in the reproductive system, abdominal and cardiac ultrasound, spinal X-ray, and bone age. She was treated with growth hormone (GH) one year ago because of her short stature. GH has been discontinued now. Because of the high risk of POI, OTC was planned. The hormone level before OTC was FSH 4.27 IU/L, LH 0.00 IU/L, E2 < 11.80 pg/ml, AMH 1.06 ng/ml. Pelvic ultrasound showed that the size of the bilateral ovaries was 1.6 cm×0.7-0.8 cm, no enlarged follicles were found, and the maximum diameter of follicles was 0.2-0.37 cm. Ovarian tissue for OTC was taken from the whole right ovary by laparoscopic surgery, and the antral follicles could be seen in ovarian tissue preparation. Sixteen ovarian cortical slices were cryopreserved by slow freezing, with an average of 1380 follicles in round cortical tissue with a diameter of 2 mm, and the follicular density was about 440/mm^3^. The ovarian tissue from 10 children with non-TS was cryopreserved in our center, the median age was 5 (range 2-8) years old, and the median number of follicles was 766 (range 163-2250) per 2 mm biopsy. The follicles number in this girl were within normal range.

**Conclusion:**

TS patients should be evaluated early in childhood to benefit from FP. For highly selected young females with mosaic TS, if the endocrine evaluation does not indicate POI and other health problems do not rule out future pregnancy, it seems reasonable to consider OTC as an FP option.

## Introduction

Turner syndrome (TS), the most common chromosome abnormality in females, is associated with the inevitable premature exhaustion of the ovarian reserve. The incidence in newborn females is about 1/4000-1/2500 (1). The pathological basis of TS is that one of the X chromosomes is intact and the other sex chromosome is entirely or partially deleted. Karyotypes 45, X account for about 50%, the mosaicism of 45, X/46, XX account for about 20%, and the rest are other structural abnormalities of the X chromosome (2). Growth retardation and gonadal hypoplasia, such as premature ovarian insufficiency (POI), are two important clinical manifestations of TS (3, 4).

The diagnosis of TS can be made by a non-invasive prenatal test (NIPT) or amniocentesis. It can also be diagnosed after birth. Peripheral blood karyotype analysis is the gold standard for diagnosing TS, which usually requires 30 standard cells. Buccal cell fluorescence *in situ* hybridization (FISH) analysis is an effective tool for analyzing and evaluating chromosome composition (5, 6). TS patients may have a variety of complications in each life stage involving multiple organs, and they require multi-disciplinary diagnosis and treatment, not only to maintain average growth and development but also to induce puberty and maintain female secondary sexual characteristics, to reach peak bone mass and to provide fertility preservation (FP). POI usually occurs before puberty in TS girls. Counseling and evaluation should be conducted as soon as possible to apply the best and most personalized FP strategies (7).

Several studies have shown that follicular dysplasia and germ cell apoptosis may be the leading causes of follicular exhaustion in TS females (8, 9). The accelerated rate of follicle loss occurs in the early stage of fetal development (10). Due to accelerated follicular atresia, most females with TS develop POI before or around puberty (11). Mosaic TS may have enough primordial follicles to experience menarche and normal puberty. Spontaneous menarche occurs in 15% of TS girls, only 2.0%~7.6% conceive naturally, and most of them are mosaic TS females (12, 13). Being unable to have a biological child is the most common and painful challenge experienced by most TS females, especially when family and friends begin to have children (14). At present, ovarian tissue cryopreservation (OTC) is the only method of FP for pre-pubertal girls (15). More than 200 healthy babies have been born through this technology worldwide (16). OTC may be a promising FP option for TS girls, with cryopreservation of the primordial follicular pool before the ovarian reserves are entirely exhausted (17).

TS patients considering pregnancy should occur only after an assessment of their cardiovascular risk by the specialist. Both natural and donated oocytes have a higher risk of pregnancy loss and obstetrical complications. Therefore, international clinical guidelines have been developed to optimize the healthcare of TS females who wish to conceive (4, 18). To improve the chances of conception, oocyte cryopreservation and OTC has been proposed for FP (19, 20). Evidence of successful FP in TS females through OTC and autologous transplantation is still lacking. More case reports and a series of reports are needed to assess the prospects of OTC in TS patients. Each case deserves to be reported to give more information and confidence about FP to young females with the same type of disease and to give doctors more knowledge about FP. And the TS community of patients, families, and healthcare providers has been waiting expectantly for reports of successful cryopreservation and FP (4, 21, 22). This study reports ovarian tissue cryopreservation for the first time applied in a 3-year-old girl with TS to preserve fertility and ovarian endocrine function in China and reviews the application and progress of FP for TS.

## Case presentation

This case presents a 3-year-old girl, height 103 cm and weight 16 kg, diagnosed with mosaic TS. Amniotic fluid puncture occurred during her mother’s pregnancy at 18 weeks, and chromosome karyotype analysis results showed 45, X[17]/46, XN[33]. The N represents X or Y because it is not allowed to determine the sex of the fetus in China. The cell culture method was routine amniotic fluid cell adherent culture. The chromosome banding method was the digestion method G-banding, and the resolution was 320 bands. The girl’s mother was 38 years old when pregnant, and a regular antenatal examination was carried out. The mother gave birth on September 28, 2018. The birth process went smoothly, and the girl had no history of hypoxia and asphyxia. The results of chromosome karyotype analysis in the umbilical cord blood showed that the karyotype was 45, X[19]/46, XX[81], and the diagnosis of TS was confirmed. The doctors recommended monitoring height, weight, and secondary sexual characteristics and follow-up in the pediatric clinic.

Over the past two years, the child was followed up in the pediatric outpatient clinic. No abnormality was found in the reproductive system, abdominal and cardiac ultrasound, spinal X-ray, and bone age. One year ago, the child was treated with growth hormone (GH) because of her short stature. Now the child is of average height and has stopped taking GH. Due to the high risk of POI, the child’s parents soon come to the FP clinic for consultation, and OTC for FP has been recommended. The patient signed the OTC agreement and gave informed consent in our center, which was approved by the Ethics Committee of the Beijing Obstetrics and Gynecology Hospital, Capital Medical University (number: 2017-KY-020-01). Written informed consent was obtained from the patient and her parents.

The girl is her mother’s second pregnancy and second delivery. Her intellectual development is the same as ordinary children of the same age, and her sister is in good health. Before OTC, the levels of endocrine hormone were as follows: anti-Müllerian hormone (AMH) 1.06 ng/ml, follicle-stimulating hormone (FSH) 4.27 IU/L, luteinizing hormone (LH) 0.00 IU/L, estradiol (E2) < 11.80 pg/ml, progesterone (Prog) 0.30 ng/ml, prolactin (PRL) 43.68 ng/ml, testosterone (T) < 6.86 ng/dl. Pelvic ultrasonography revealed that the uterine length was 1.9 cm, the anteroposterior diameter was 0.4 cm, and the cervical length was 1.7 cm. The size of the left ovary was 1.6 × 0.7 cm, and there was no enlarged follicle, the maximum diameter of the follicle was 0.2 cm. The right ovary size was 1.6 × 0.8 cm, there was no enlarged follicle, and the maximum diameter of the follicle was 0.37 cm.

**Figure 1:**
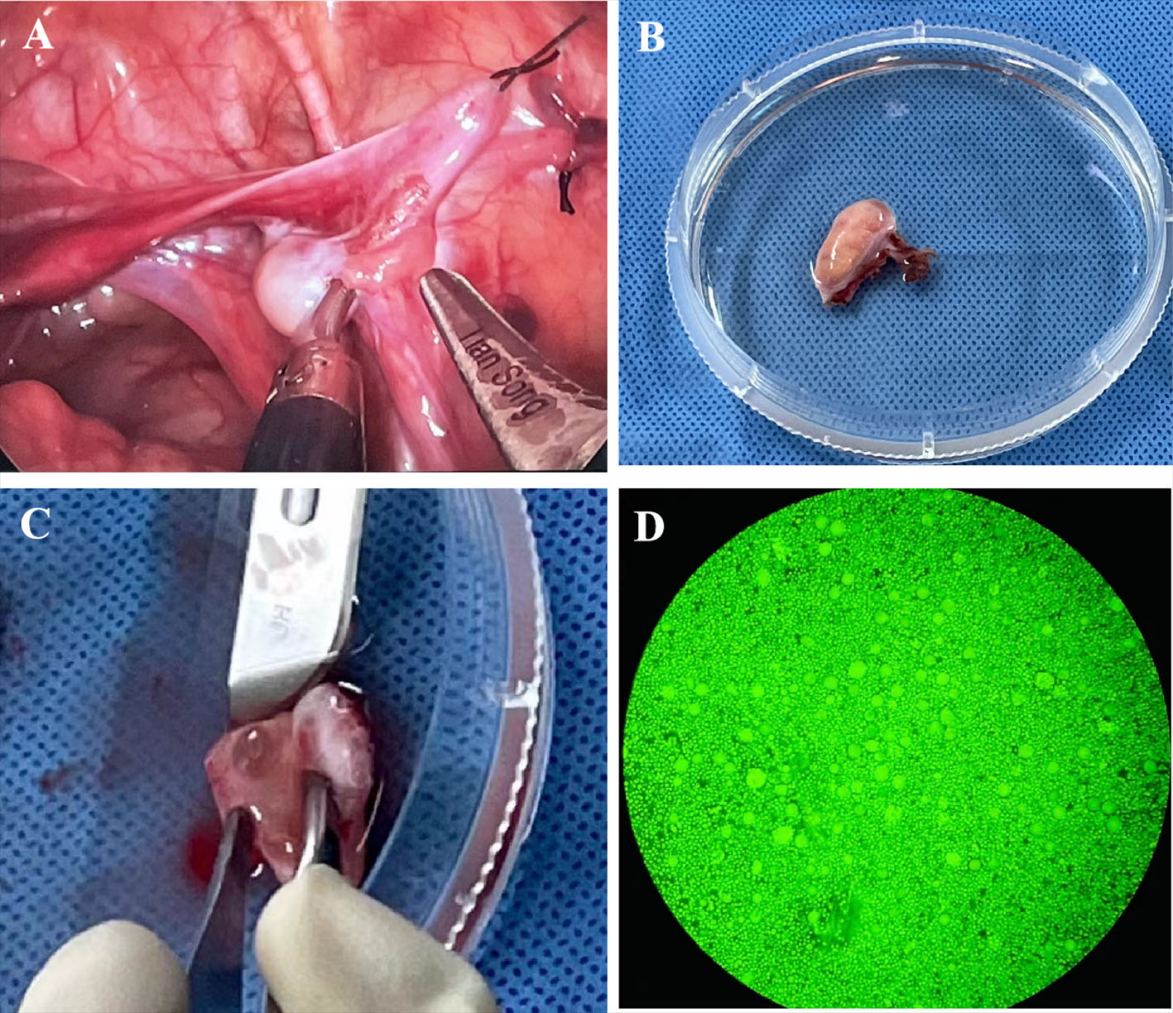
**(A)** Photos of the ovary during ovarian tissue biopsy; **(B)** Ovarian tissue was transported to ovarian tissue cryobank for ovarian tissue preparation; **(C)** An antral follicle can be seen during ovarian tissue preparation; **(D)** Detection of follicular activity in ovarian cortex.

**Figure 2:**
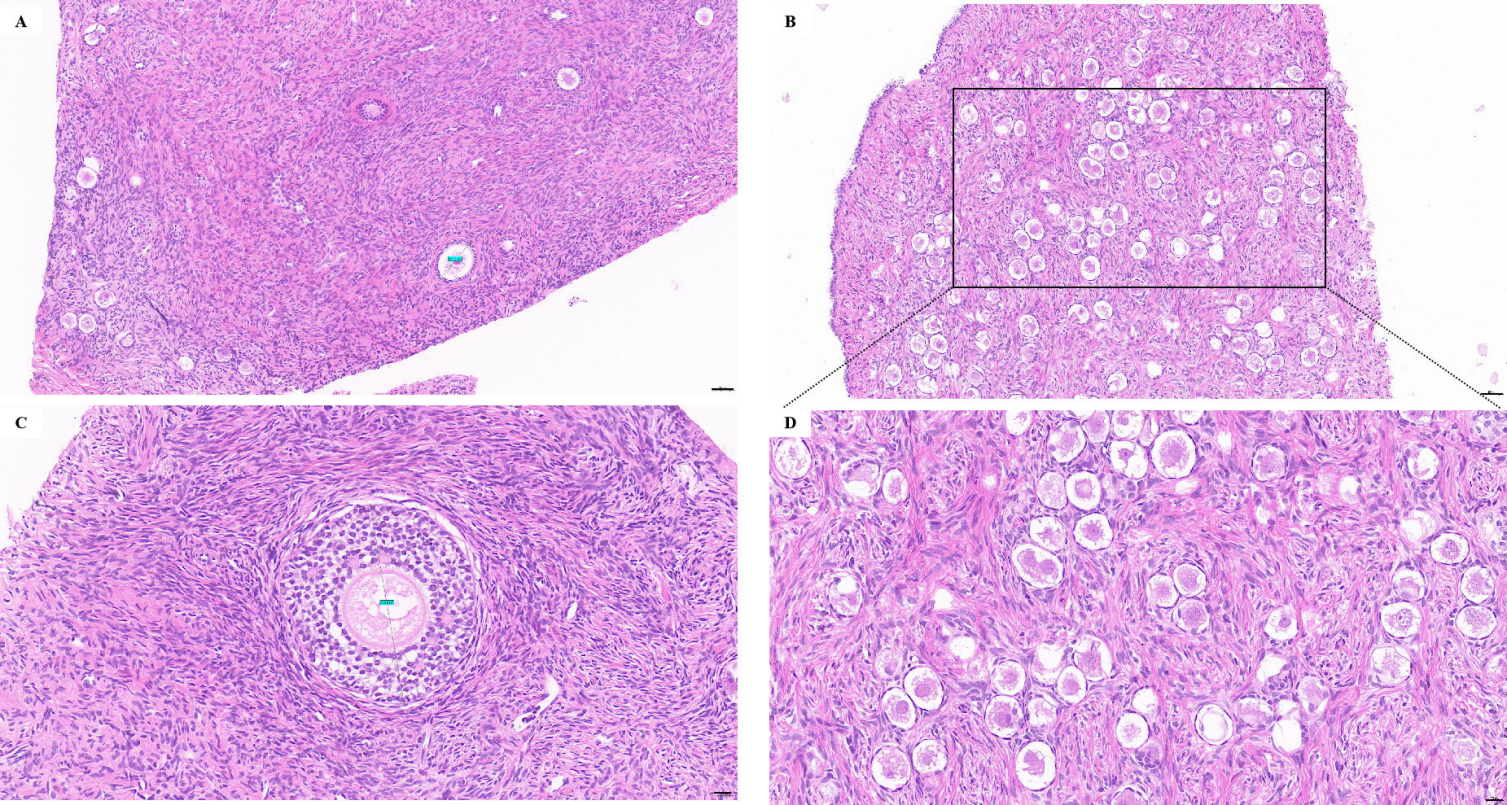
**(A, C)** The typical pictures of HE staining of the ovarian cortex of this TS girl can see the different stages of follicles, most of which are primordial follicles; **(B, D)** The typical pictures of HE staining of non-TS girl. Bars: **A**, **B** = 50 μm, **C**, **D** = 20 μm.

The whole right ovary was removed at Children’s Hospital, Capital Institute of Pediatrics, on April 29th, 2022, and normal ovarian morphology could be observed (**Figure 1A**). The ovarian tissue was quickly placed in a low-temperature transfer solution *in vitro* and transported to our ovarian tissue cryobank within 1 hour (**Figure 1B**). During the preparation of ovarian tissue, the follicles reported by ultrasound were found (**Figure 1C**). The ovarian tissue was divided into 16 cortical slices with thicknesses of 1 mm and 3 mm x 6 mm in width and length. Round cortical slices of standardized size (diameter 2 mm) were taken from the remaining cortex to evaluate follicle number. The method of follicle counting was the same as the previously published paper (23), with an average of 1380 follicles in a diameter of 2 mm of the round cortical slice, and the follicle density was about 440/mm^3^ (**Figure 1D**). Ten children with non-Turner syndrome diseases were cryopreserved ovarian tissue in our center. The median age was 5 (range 2-8) years old, and the median number of follicles was 766 (range 163-2250) per 2 mm biopsy. The typical pictures of hematoxylin-eosin (HE) staining of the ovarian cortex of this TS girl can see the different stages of follicles, most of which are primordial (**Figures 2A, C**). The typical HE staining pictures of non-TS girls are shown in **Figures 2B, D**.

## Discussion

To the best of our knowledge, this is the first case report of successful cryopreservation of ovarian tissue in patients with TS in China. Young females who need gonadotoxicity therapy have an increasing demand for FP. TS has been recognized as the most established genetic cause of POI and usually occurs before puberty (24). It is suggested that the accelerated apoptosis of germ cells may be the primary mechanism of follicular depletion in TS (6). The possibility of ovarian function in patients with TS depends on the presence of 46, XX germ cells in the ovaries. Girls with mosaic TS are more likely to retain spontaneous puberty, menarche, and fertility than complete 45,X (13).

The advances in cryopreservation technology have raised hopes of FP in women at risk of POI. High-quality FP counseling includes fertility consideration, reproductive health protection, future health consequences, and FP outcomes (25, 26). The best choice should be determined not only based on individual ovarian reserves but also on genes and heart health status (27). FP in each TS case should be evaluated individually. The maternal risk of TS females includes maternal mortality during pregnancy, mainly due to cardiovascular risk, which must be considered when planning a pregnancy in TS patients (28–30). It should be considered that there are only a few reports of live births using frozen oocytes in TS females.

According to the psychosocial maturity of TS adolescents, it is generally recommended that FP be considered and discussed early after menarche. For FP, it is reported that oocytes are frozen after ovarian stimulation shortly after puberty (31, 32). It is recommended that a series of anti-Müllerian hormone (AMH) assessments are performed to determine the best time for intervention, i.e. before AMH reaches a plateau and begins to decline (32). Ovarian stimulation should not be postponed to ensure optimal ovarian response and maximize the number of oocytes, especially considering preimplantation genetic testing for aneuploidy (PGT-A) when using oocytes (33).

However, unfortunately, only 5~20% of women with TS undergo menarche (11, 34). Even candidates for oocyte cryopreservation may be unsuccessful in freezing mature oocytes. Therefore, physicians should consider the pros and cons when providing FP consulting to girls and young females with TS. Because of the high risk of POI, timely consulting about FP options is essential. An interview study showed that, regardless of age, uncertainty about their fertility is one of the major concerns for girls and females with TS and their parents (14).

So far, no study has reported the relationship between TS mosaic levels and ovarian reserve biomarkers (AMH and antral follicle counting, AFC). Women with mosaic TS are also likely to develop accelerated follicular loss, and there is no reliable phenotypic correlation (35). The phenotypic characteristics of patients with mosaic TS are usually not prominent, and the risk of fertility and pregnancy is also better than that of women with X chromatid. It was reported that in X-chromatid women, the levels of AMH decreased significantly, and mosaic and structural abnormalities slightly decreased (36). If AMH is not detected, FSH levels rise, and antral follicles and ovarian cords are absent, which indicates POI, it highly unlikely that oocyte cryopreservation or OTC would be successful.

Only very recently (2022), cryopreservation of oocytes after ovarian stimulation and the successful delivery of a healthy female baby by PGT-A in a mosaic TS patient after menarche was reported in Belgium (5). Oocyte vitrification after controlled ovarian stimulation (COS) does not reduce the lifespan of natural ovaries, but this technique can only be provided to natural post-pubertal girls (19). However, it has been reported that the oocytes of a pre-pubertal girl have been successfully cryopreserved. The patient was a 7-year-old girl with mosaic TS, 45, X [37]/47, XXX [19]. After a short course of ovarian stimulation with recombinant gonadotropins (FSH and LH) and human chorionic gonadotropin (hCG), six metaphase II (MII) oocytes were successfully obtained, bypassing the physiological process of the hypothalamus-pituitary-ovary (HPO) axis during puberty and directly targeting the ovaries to obtain mature follicles (37).

In summary, mature oocytes cryopreservation is limited to a small percentage of TS females, those who will be fertile after the spontaneous onset of puberty and menstruation. Furthermore, the patient has to be emotionally enough to undergo the ovarian stimulation with exogenous FSH administration followed by transvaginal ultrasound-guided oocyte retrieval (18).

Some studies have shown that despite the normal morphology of ovaries and follicles, stromal cells and granulosa cells of small follicles in patients with TS may show a high degree of mosaicism (18, 20). In addition, the mosaicism level of ovarian cells cannot be predicted from the analysis of extra-ovarian tissues. Furthermore, 45, X karyotype in peripheral blood cells cannot rule out the existence of 45, X/46, XX mosaicism in the ovary (38). Recently, Schleedoorn et al. highlighted the practical dilemma (i.e., to perform OTC surgery in a 45, X TS patient or not) by reporting on the presence of follicles in a 13-year-old female diagnosed with 45, X monosomy and an unmeasurable AMH (39).

To our knowledge, despite the reports of OTC, no one has transplanted cryopreserved ovarian tissue back to TS patients. Since 2002, more than 100 young women with TS have undergone OTC in Denmark, the United Kingdom, Australia, et al. (20, 40–44). There is a lack of the best identification index for the presence or absence of follicles. It is generally believed that mosaicism is the most promising group to have ovarian follicles and benefit from FP. The next step in the future study is exploring the effectiveness and safety of TS female FP, including pregnancy rate, outcome, and long-term follow-up. The Turner Fertility Study was initiated at the Radboud University Medical Center in the Netherlands in 2017 (45).

OTC technology was no longer considered experimental technology by ASRM in 2019, and more than 200 babies have been born through this technology worldwide (16, 46). These include OTC in childhood and then transplanting the ovarian tissue back into the body after adulthood to give birth to the baby successfully (47, 48). OTC is an established method of FP, which can preserve the fertility of young females facing iatrogenic POI or with a high risk of POI because of genetic (49). Our center has successfully cryopreserved more than 400 cases of ovarian tissue, and 10 cases have been successfully transplanted. The ovarian function has been restored 2 to 4 months after ovarian tissue transplantation (OTT) (50). One patient delivered a healthy baby girl, the first baby born through OTC and transplantation technology in China (51). Thirty-five medical experts pointed out that OTC should only be provided for TS patients in a safe and controlled research environment (45, 52). Since TS patients usually have fewer ovaries and are younger when performing OTC, it is usually recommended that unilateral whole ovarian tissue is obtained (38). OTC can be provided to TS girls whose ovaries have sufficient reserves but cannot wait until they are mature enough for oocyte cryopreservation.

In this case report, the follicular density of the TS girl was within the normal range. One study has shown that the ovarian tissue evaluation of 15 TS patients aged 5 to 22 years old showed that follicles could be seen in 60% of the ovarian tissues, 78% of the follicular density in the ovaries with follicles was within 95% confidence interval of the control group, and the rate of follicular abnormality was high. The benefits of OTC may be limited to a group of highly selected patients with mosaic TS in which there is a large normal follicular pool at OTC. Still, there have been no clinical data about OTT in mosaic TS patients until now to support it (20, 53). Immature oocytes were obtained from unstimulated small antral follicles in the ovarian tissue, followed by *in vitro* maturation (IVM) in which oocytes are cultured from the germinal vesicle (GV) to the MII stage and then cryopreserved, increasing the FP potential of patients, even though the poor yield is expected (42, 54).

## Limitation

In this study, FISH karyotype analysis was not carried out in the ovarian tissue of the patient to further analyze the mosaicism of 46, XX in oocytes, granulosa cells, and stromal cells. Because aneuploidy in granulosa cells and oocytes may disrupt the normal development of follicles, which could harm the success rate of auto-transplantation of ovarian cortex tissue in the TS patient group, we will do the FISH karyotype analysis in the frozen-thawed ovarian cortex before ovarian tissue transplantation.

## Conclusion

In summary, TS girls have a very high risk of POI and infertility. TS patients should be evaluated in early childhood to benefit from FP because most girls’ ovarian reserves run out before and around puberty. For highly selected adolescent patients with mosaic TS, if the endocrine evaluation does not indicate POI and other health problems do not rule out pregnancy, it seems reasonable to consider OTC to preserve fertility and ovarian endocrine function. Frozen-thawed ovarian tissue transplantation has not been carried out in patients with TS, and whether it can restore female fertility remains to be followed. Healthcare providers should positively refer young girls with TS to FP clinics for FP consulting, which is essential for TS patients.

## Author contributions

JC wrote the original draft, revised the manuscript, and transported and cryopreserved the ovarian tissue. XR acquired the medical report and data from the patient, project, and funds leader. JD, FJ, and MG cryopreserved the ovarian tissue. YW ovarian tissue biopsy. AM guided the implementation of the project and revised the final manuscript. All authors contributed to the article and approved the submitted version.

## Funding

This study was supported by Natural Science Foundation of Beijing (7202047), Beijing Capital Foundation for Medical Science Development and Research (2020-2-2112), and the Beijing Municipal Administration of Hospitals’ Ascent Plan (DFL20181401).

## Conflict of interest

The authors declare that the research was conducted in the absence of any commercial or financial relationships that could be construed as a potential conflict of interest.

## Publisher’s note

All claims expressed in this article are solely those of the authors and do not necessarily represent those of their affiliated organizations, or those of the publisher, the editors and the reviewers. Any product that may be evaluated in this article, or claim that may be made by its manufacturer, is not guaranteed or endorsed by the publisher.
